# Analysis of pregnancy outcome after anastomosis of oviduct and its influencing factors

**DOI:** 10.1186/s12884-019-2469-2

**Published:** 2019-10-30

**Authors:** Yun Feng, Han Zhao, Hongxia Xu, Ying Ai, Lingyun Su, Li Zou, Linna Yang, Dehong Yang, Xuelan Yan, Na Ma, Wei Dong

**Affiliations:** 1 Department of Obstetrics and Gynaecology, The First People’s Hospital of Yunnan Province, No.157 of Jinbi road, Kunming, 650032 China; 2 Medical School of Kunming University of Science and Technology, Kunming, 650500 China

**Keywords:** Laparoscopic, Oviduct anastomosis, The pregnancy rate, Correlated factors

## Abstract

**Background:**

This study aims to investigate the influencing factors of pregnancy after laparoscopic oviduct anastomosis.

**Methods:**

The data of 156 cases of laparoscopic oviduct anastomosis in our hospital were analyzed.

**Results:**

The pregnancy rate decreased with age (*P* < 0.005). The pregnancy rate after six years of anastomosis was higher in those with ligation (*P* < 0.005). The postoperative pregnancy rate significantly increased in subjects with oviduct lengths of > 7 cm (*P* < 0.01). The pregnancy rate of isthmus end-to-end anastomosis was higher (*P* < 0.005). The pregnancy rate after bilateral tubal recanalization was higher than that after unilateral tubal recanalization (*P* < 0.005). The pregnancy rate after laparoscopic tubal ligation and laparoscopic anastomosis was higher than that of open tubal ligation and laparoscopic anastomosis (*P* < 0.005).

**Conclusion:**

The pregnancy rate after laparoscopic oviduct anastomosis is higher in subjects below 35 years old, with a ligation duration of < 6 years, and a length of oviduct of > 7 cm, and those who underwent isthmus anastomosis and laparoscopic oviduct ligation and recanalization.

## Background

With the full relaxing of China’s second-child policy, more women who underwent tubal oviduct ligation want to recanalize the oviduct and get pregnant again. Thus, they face a choice of reoperation for oviduct recanalization. Oviduct anastomosis is an operation to reconstruct the structure and function of the oviduct after tubal ligation [1], and has been proved to be effective in younger women [2].

The clinical data of 156 women after oviduct anastomosis in our hospital were retrospectively analyzed. The factors that influenced pregnancy outcome, in terms of the age of the recipient, the duration of the sterilization period, the length of the oviduct after recanalization, and the method of oviduct anastomosis and previous tubal ligation method, were investigated to provide the basis for evaluating the indications of oviduct anastomosis in clinical application.

## Methods

### General information

A total of 156 subjects, who underwent laparoscopic oviduct anastomosis in our department from January 2014 to October 2018, were enrolled as the study subjects. All subjects had a regular menstrual cycle. The age of these subjects ranged within 23–45 years old, with an average age of 35.04 years old. The duration of the sterilization period of these subjects ranged within 1–23 years, with an average duration of 8.77 years. Among these subjects, 92 subjects underwent open oviduct ligation, while 64 subjects underwent laparoscopic oviduct sterilization. All subjects were assessed for normal ovarian function and menstrual cycle before the operation. Subjects with uterine and appendage tumors were excluded. No surgical contraindication was found in these subjects. Furthermore, the semen examination results of their spouses were normal.

### Surgical methods

Selection of time of operation: The operation was performed on the 3-10th day after the menstruation stopped. Surgical procedures: All subjects underwent laparoscopic oviduct anastomosis. Anesthesia procedure: Subjects were treated with general anesthesia and endotracheal intubation. Surgical methods: Subjects were placed in the lithotomy position, routine disinfection was performed, and the uterine cavity dual-lumen tube was placed for laparoscopic oviduct hydrotubation. The umbilicus was taken as the first puncture hole. The skin and part of the fascia was cut. A 10-mm puncture device was punctured in. CO_2_ was used to form pneumoperitoneum. A laparoscope was placed to explore the abdominopelvic cavity. Two 5-mm incisions were made at the skin 5 cm to the lower left from the scar of original operation and theumbilicus. The 5-mm puncture device was inserted into the abdominal cavity. The uterus, pelvic cavity and bilateral ovaries were observed. If lesions were present, these were treated, accordingly. The diluted methylene blue solution was injected through the double-lumen tube, and the oviduct sites where the methylene blue solution flowed through were observed. The serosal layer of the oviduct was incised according to different ligation sites. The bilateral broken ends of oviducts were freed. The scar tissues at the broken ends were cut off. Electrocautery was performed at the bleeding point. For subjects who underwent laparoscopic oviduct ligation with a titanium clip, the titanium clip was removed, and the closed ends of oviduct were cut off. Methylene blue solution was injected through the vaginal dual-lumen tube. It could be observed under a laparoscope that the methylene blue solution flowed smoothly from the proximal end. For this case, the distal oviduct lumen was positioned. Otherwise, an epidural catheter was sent to pass through the oviduct fimbria to the incision site, and three stitches of intermittent full-thickness suture of both broken ends of the oviduct were performed with 5/0 absorbable surgical suture. Then, the mesosalpinx was sutured.

### Criteria for determining the patency of the oviduct after anastomosis

After performing the hydrotubation again, the methylene blue solution smoothly flowed out the oviduct fimbria. The length of the oviduct was not less than 6 cm. If a small volume of methylene blue solution leaked out from the anastomosis site, reinforcement and suture were not required, since this would not affect the healing of the anastomosis and the pregnancy rate after the operation [2]. A small volume of normal saline (N.S) was injected into the pelvic cavity to keep the oviduct wet during the operation.

### Clinical curative effect evaluation

At one month after the operation, the subjects were guided to naturally conceive and underwent followed ups. The pregnancy of subjects after the operation was recorded, and the factors that affected the pregnancy were analyzed.

The diagnostic criteria of pregnancy include: (1) dysmenorrhea; (2) blood test was positive for Human Chorionic Gonadotropin (HCG) and gynecological b-ultrasound indicated intrauterine pregnancy.

### Statistical methods

The study data were statistically analyzed using statistical software SPSS 11.0. Intergroup comparison of count data was performed using *X*^2^-test.

## Results

### Surgical outcomes

The bilateral oviducts became unobstructed in 127 subjects, while a single side of the oviduct became unobstructed in 29 subjects, and the patency rate was 100%.

### Pregnancy of subjects after the operation

A total of 106 subjects got pregnant after the recanalization, accounting for 67.94%. Among these subjects, 99 subjects (99/127) got pregnant after bilateral oviduct recanalization, and the pregnancy rate was 77.95%, while seven subjects (7/29) got pregnant after the unilateral oviduct recanalization, and the pregnancy rate was 24.14% (*P* < 0.005). Furthermore, 54 subjects (54/92) who previously underwent open laparotomy oviduct ligation got pregnant after recanalization, and the pregnancy rate was 58.7%, while 52 subjects (52/64) who previously underwent laparoscopic oviduct ligation got pregnant after recanalization, and the pregnancy rate was 81.2% (*P* < 0.005). Moreover, three subjects (3/11) with an oviduct length of less than 7 cm got pregnant after anastomosis, and the pregnancy rate was 21.3%, while 103 subjects (103/145) with an oviduct length of greater than 7 cm got pregnant after anastomosis, and the pregnancy rate was 71.0% (*P* < 0.01) (Tables 1, 2 and 3).

**Table 1:** C pregnancy rate after tubal anastomosis and the age of patient

Age (year)	Number of case	Rate of pregnancy(%)
≦35	84	76.19%(64/84)χ2:8.32: *P* < 0.005
36–40	56	67.85%(38/56)χ2:12.89: *P* < 0.005
> 40	16	25%(4/16)
Total	156	67.94%(106/156)

**Table 2:** Relationship between sterilization duration and pregnancy rate after anastomosis

Duration (year)	Number of case	Rate of pregnancy(%)
<6	37	81.08%(30/37) χ2:12.59: *P* < 0.005
6–10	73	79.45%(58/73) χ2:14.35: *P* < 0.005
11–14	30	46.66%(14/30) χ2:1.66: *P* > 0.1
> 15	16	25%(4/16)
Total	156	67.94%(106/156)

**Table 3:** Relationship between anastomotic site and pregnancy rate

Site	Number of case	Rate of pregnancy(%)
isthmus-isthmus	81	85.18%(69/81)
isthmus-ampulla	27	66.66%(18/27)
ampulla-ampulla	48	39.58%(19/48) χ2:20.21: *P* < 0.005
Total	106	67.94%(106/156)

## Discussion

In the present study, the data revealed that the re-pregnancy rate reached 76.19% in subjects with an age of ≤35 years old after oviduct anastomosis, and this was higher than that in the other two groups (*P* < 0.005). This result suggests that the older the recipient, the lower the rate of re-pregnancy after anastomosis [3]. Therefore, the age at the time of recanalization is correlated to the postoperative pregnancy rate. Hence, it is particularly important to take into account both effective sterilization and the possibility of re-pregnancy [4]. However, age can affect fertility rates. So we should compare the data in our study with the data of the patients that are at the same age range of the patients in our study, but did not do laparoscopic oviduct anastomosis, so as to eliminate the effects of age.

The difference in length of the oviduct after oviduct anastomosis also directly affected the re-pregnancy rate of the subjects. The pregnancy rate was 71.0% in subjects with an oviduct length of ≥7 cm. This was significantly higher than that in subjects with an oviduct length of < 7 cm (*P* < 0.01), and the difference was statistically significant. An oviduct length of < 5 cm can directly affect the fertilization and delivery of fertilized eggs, leading to a lower pregnancy rate. A short oviduct may cause these fertilized eggs to enter the uterine cavity too early, in which the development does not synchronize with the endometrial development, leading to spontaneous abortion or biochemical pregnancy [5].

In the present study, the re-pregnancy rate was 85.18% in subjects after isthmus-ampulla anastomosis of the oviduct, and this was significantly higher than that in the other groups, and significantly higher than that in subjects receiving ampulla-ampulla anastomosis of the oviduct (39.58%, *P* < 0.005). A study reported that the re-pregnancy rate was higher in subjects after isthmus-isthmus anastomosis of the oviduct, when compared to that of subjects after anastomosis at other sites of the oviduct [6]. After the isthmus-ampulla anastomosis of the oviduct is performed, since the ampulla is rich in wrinkles, some oviduct mucosal wrinkles would often protrude outside of the anastomosis, which may affect the healing of the anastomosis, and subsequently affect the surgery [7].

The pregnancy rate after recanalization was higher in subjects who previously underwent laparoscopic oviduct ligation, when compared to subjects who previously underwent open oviduct ligation (*P* < 0.005). This was correlated to the characteristics of laparoscopic surgery, such as small incision, less abdominal interference, less tissue damage, and safety under direct vision [8]. A related study revealed that the incidence of adhesion after laparoscopic surgery was lower than that after open surgery [9]. Subjects who underwent oviduct ligation with a titanium clip had a longer retained segmental length and less damage to the oviduct. Oviduct lumen adhesion, stiffness, or traction, and the distortion or occlusion of the surrounding scar tissues [10] may cause the oviduct to lose its physiological function, such as the transport of sperm and eggs, and the fertilization of eggs.

At present, traditional open oviduct anastomosis, transabdominal microscopic oviduct anastomosis and laparoscopic oviduct anastomosis are the clinical methods of oviduct anastomosis. Microscopic oviduct anastomosis can anastomose the oviduct in a full-thickness, accurate, multi-needle and clear manner. Furthermore, the stent may not be placed, and fine needle and fine thread can be used to eliminate the stimulation and injury of foreign bodies to the oviduct mucosa, which practically avoids scars. Therefore, traditional concepts consider that only open surgery under a microscope can lead to a good prognosis [11–13]. However, with the improvement of laparoscopic microdevices and development of technology, an increasing number of literatures on laparoscopic oviduct anastomosis have been reported in recent years [14–16]. On a laparoscopically magnified TV screen, the cut ends of the oviduct are clearly displayed, making the oviduct anastomosis simple and easy under a laparoscope. Furthermore, the involution is good, and the characteristics of the laparoscopic operation, such as precision, meticulousness and accuracy, are fully applied. The oviduct mucosa is one of the most vigorously growing tissues among human tissues. Therefore, there is no need for excessive suture during surgery. In principle, as long as the lumens of the two ends are accurately aligned and the thickness is basically the same, the purpose can be achieved [17]. During the operation, attention should be given to the misalignment of the cut ends of the oviduct, which cannot be misplaced. If the diameter of the proximal end lumen is smaller than the distal end, the proximal oviduct can be cut in chamfer to preserve the oviduct tissue, as much as possible [18]. Furthermore, attention should be given to ensure that sufficient mesangial covers the surface of the wound of the oviduct after the anastomosis, in order to prevent adhesion formation [19].

Our strengths are that the sample size was not small, thus the result was convincing, and we found out some factors that can affect the effects of laparoscopic oviduct anastomosis, for example, ligation duration and length of oviduct. However, age per se can affect fertility rates, but we did not compare the data in our study with the data of the patients that are at the same age range of the patients in our study, but did not do laparoscopic oviduct anastomosis, which should be addressed in future studies. In addition, we did not compare the effects of laparoscopic oviduct anastomosis with in-vitro fertilization (IVF). Also, we did not evaluate the postoperative complications, which can be discussed in future studies. In addition, due to the long duration of pregnancy and limited trial time, the pregnancy outcomes of patients after fallopian tube anastomosis are still being followed up and no systematic data have been obtained, which can be mainly discussed in future studies. We have added it as a limitation of our study in the discussion section.

## Conclusion

In summary, compared with open surgery, laparoscopic oviduct anastomosis has a clearer field of vision, more accurate involution and less interference, and the rate of re-adhesion is lower. However, there are many factors that affect the postoperative pregnancy of subjects. In addition, before surgery, the patient’s age, duration of sterilization period and ovarian function should be fully evaluated, and the surgical actions need to be gentle, careful and accurate. Furthermore, the tissues separated and sutured during surgery should be as few as possible, and attention should be given to reduce injury to the mesangium, as much as possible, during surgery. When the serosa is sutured, care should be given to avoid twisting the oviduct. In addition, attention should be given to avoid damage to the oviduct cilia and anastomosis tissues, and reduce the scar formation of anastomosis, in order to facilitate the passage of fertilized eggs and improve the pregnancy rate.

## Data Availability

The datasets used and/or analysed during the current study available from the corresponding author on reasonable request.

## Abbreviations

HCG: Human Chorionic Gonadotropin
IVF: in-vitro fertilization
N.S: normal saline
